# No effect of mate novelty on sexual motivation in the freshwater snail *Biomphalaria glabrata*

**DOI:** 10.1186/1742-9994-6-23

**Published:** 2009-10-12

**Authors:** Ines K Häderer, Johanna Werminghausen, Nico K Michiels, Nadine Timmermeyer, Nils Anthes

**Affiliations:** 1 Animal Evolutionary Ecology, Institute for Evolution and Ecology, Eberhard Karls-Universität Tübingen, Auf der Morgenstelle 28, 72076 Tübingen, Germany

## Abstract

**Background:**

When mating effort (e.g. via ejaculates) is high, males are expected to strategically allocate their resources depending on the expected fitness gains from a given mating opportunity. One mechanism to achieve strategic mating is the Coolidge effect, where male sexual motivation declines across repeated encounters with a familiar partner, but resuscitates when encountering a novel female. Experimental tests of male mate choice via mechanisms such as the Coolidge effect, however, remain scarce. Moreover, it is untested to date whether the Coolidge effect occurs in a sex-specific manner in simultaneous hermaphrodites, where the motivation to mate with a familiar partner may vary with previous mating activity in the male or female role.

**Results:**

We exposed focal hermaphroditic freshwater snails, *Biomphalaria glabrata*, repeatedly to either a familiar or a novel partner. None of our proxies of sexual motivation (remating likelihood, mating delay, copulation duration) varied between the novel and familiar partner treatments. Moreover, the mating role taken during the first copulation did not affect the subsequent choice of mating roles in the familiar partner treatment as would be expected if focals preferred to avoid mating twice in the same role with a familiar partner. This indicates the absence of sex-specific effects of partner novelty.

**Conclusion:**

Our data indicate that mate novelty does affect neither overall sexual motivation nor the choice of mating roles in *B. glabrata*. Hence, male mate choice via a Coolidge effect appears inexistent in this invertebrate hermaphrodite. We discuss the possible roles of insufficient fitness gains for discriminatory behaviour in populations with frequent mate encounters as well as poor mate discrimination capacities. Our findings lend also no support to the novel prediction that sexual motivation in simultaneous hermaphrodites varies with the mating roles taken during previous copulations, calling for empirical investigation in further hermaphrodite systems.

## Background

While the metabolic costs of producing a single sperm cell are almost negligible, the total paternal investment in sperm and seminal fluid that is required to sire a single offspring may often match the maternal per egg investment [1-4]. Males thus benefit from carefully choosing their mating partners and prudently adjusting mating effort (e.g., ejaculate size) to the expected gain in reproductive success. Even though mechanisms of male mate choice are subject to rising attention (reviewed in [5-7]), empirical assessments remain scarce compared to the large body of literature devoted to female mate preferences. Emerging evidence now indicates that male mating propensity and sperm allocation to individual partners can indeed vary in response to female fecundity or the risk of sperm competition in fruit flies [3,8], crickets [9-11], butterflies [12], or birds [13].

An additional way in which males could strategically distribute sperm is by preferring copulations with novel mates over copulations with familiar partners. This should be beneficial under three conditions [6]. First, frequent mating exhausts male sperm stores such that males that multiply inseminate the same female risk sperm depletion upon their next encounter with a novel female. Second, repeated inseminations of the same female generate diminishing fitness returns for sperm investment due to increased competition among this male's sperm. The latter excludes cases where high 'sperm doses' within the female reproductive tract are necessary to achieve fertilisation success [14]. Third, the likelihood of encountering a novel partner is sufficiently high. Then, males should deliver smaller ejaculates in consecutive inseminations of a given female to conserve sperm reserves for future matings with novel partners that offer higher fecundity potential [6], and thus seek opportunities to secure a novel mate.

A proximate mechanism to mediate such a strategy is a decline in male sexual motivation when encountering familiar partners [15]. This process, often referred to as the Coolidge effect [16], has originally been defined when sexual satiation with a familiar female is followed by a restoration of male mating behaviour after exposure to a novel female [17]. Subsequent studies specified further proxies for 'satiation' or a 'restoration of mating behaviour' [6,18]. Accordingly, the Coolidge effect could manifest via (i) mate rejection, (ii) increased reluctance to mate, or (iii) the donation of smaller ejaculates when encountering a familiar partner.

The Coolidge effect requires an ability to differentiate between familiar and novel sexual partners. Since such cognitive capacities may primarily be expected in vertebrates, it comes as no surprise that experimental tests have some tradition primarily in rodents and livestock [17,19-24]. Only few studies to date explicitly assessed the Coolidge effect in invertebrates: In the hermaphroditic freshwater snail, *Lymnaea stagnalis*, Koene & Ter Maat [25] found a higher incidence of remating in focal individuals that were confronted with a novel rather than with a familiar partner. Likewise, Steiger et al. [26] showed that sexual motivation in the burying beetle, *Nicrophorus vespilloides*, is restored if an unfamiliar female is introduced to a male that had been exposed four times to a familiar female. In the cricket, *Gryllus bimaculatus*, Bateman [27] showed that the Coolidge effect is by no means restricted to males, but can also be expressed by females. This is beneficial whenever polyandrous matings enhance female fitness relative to multiple matings with the same male [18,28]. A similar discriminatory behaviour is shown by female decorated crickets, *Gryllodes sigillatus *[29], where a follow-up study showed that mate choice via a Coolidge effect is only expressed in females and not in males [30]. Some further studies, while not explicitly addressing the Coolidge effect, documented patterns that match a scenario of restored male (or female) sexual motivation when encountering unfamiliar partners [31-35]. Taken together, these studies suggest that the Coolidge effect may be more prevalent among invertebrates than previously thought, but generalisations require studies from a greater diversity of taxa.

Simultaneous hermaphrodites can express the Coolidge effect in an intriguing additional way. Here, sexual motivation may vary with the mating roles adopted during previous copulations. Imagine a focal individual that encounters a familiar partner with which it previously mated in the female role. The focal should now be less motivated to take the female role a second time, but highly motivated to adopt the male role. The reverse pattern of sexual motivation is expected in the partner, predicting that mating roles are alternated between the first and second copulation. In contrast, focals encountering a novel partner should display high motivation to exhibit both sex functions (with the same being true for their partner), predicting random choice of mating roles. Analysing the choice of mating roles as a function of partner familiarity thus represents a novel approach to complement previous explanations for the variable patterns of sex role alternation displayed by simultaneous hermaphrodites (e.g. [36-40]).

In the current study we seek hints for a Coolidge effect in the tropical pulmonate freshwater snail *Biomphalaria glabrata*. This preferentially outcrossing simultaneous hermaphrodite inhabits small streams and ponds in large populations [41] where familiar partners could be rejected at low costs. The typically unilateral copulations [42] are initiated when a male actor mounts the shell of a prospective mate. The male actor then moves towards the frontal left edge of the partner's shell, where it probes the female gonopore with its penis to subsequently achieve penis intromission. Following a typically 5-87 *min *penis intromission with usually successful sperm transfer [43], the male actor retracts to terminate copulation. Mating roles are subsequently exchanged in about 45% of all copulations, with the male actor now taking the female role, and *vice versa*. In non-manipulated laboratory observations, individual *B. glabrata *did not exhibit any preference to copulate in the male or female mating role, nor were there indications for non-random alternation of mating roles [44] as would be expected in systems with conditional reciprocity [45-47]. Nonetheless, given that excess sperm are often digested rather than used for fertilisation [48,49], individuals would clearly benefit from retaining costly sperm and seminal fluid for novel rather than familiar partners.

**Figure 1:**
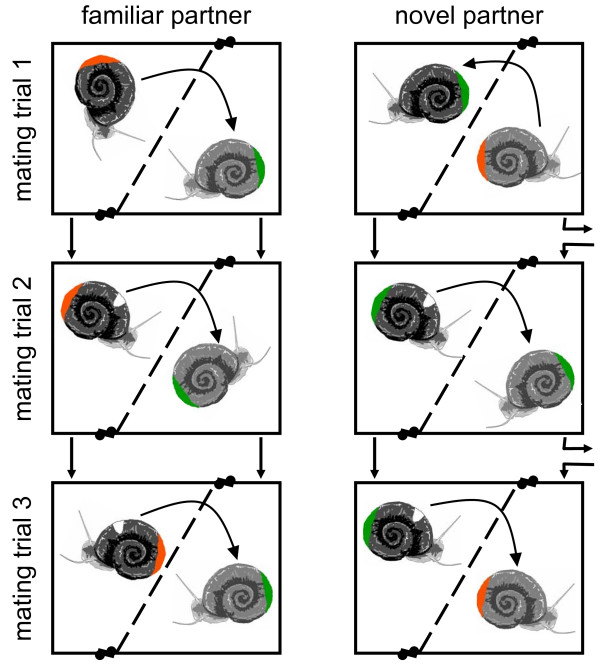
**Experimental setup illustrating the two treatments and three successive mating trials**. Green and orange labels served to distinguish snails during the first mating trial and were applied randomly within pairs. The male actor from the first mating trial was defined 'focal' and marked with an additional white dot. Curved arrows indicate which of the two snails was transferred to the partner's compartment (random during first mating, the focal in both other matings). The small arrows between observation boxes show whether pairs stayed together in the same constellation ('familiar partner') or whether the focal individual obtained a new partner for each mating ('novel partner').

To detect changes in sexual motivation as predicted by the Coolidge effect, we first measured remating likelihood, the delay until penis intromission, and intromission duration in focal snails consecutively confronted with either novel or familiar partners (see Fig. 1 and Methods section). Second, we related male sexual motivation to the mating roles adopted during previous copulations. Hence, for the first time, we investigated the interaction between the choice of mating roles and the Coolidge effect in a simultaneous hermaphrodite.

## Results

### Mate novelty and sexual motivation

**Figure 2:**
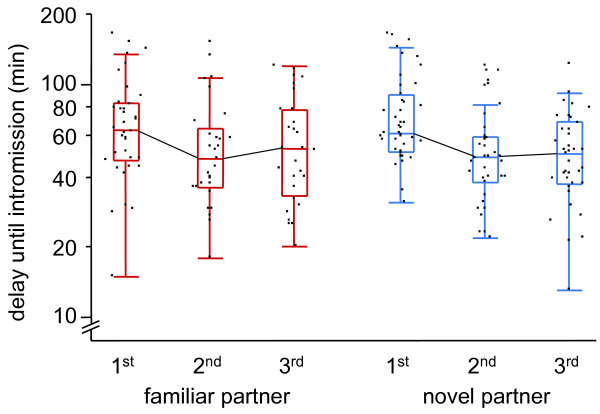
**Delay until penis intromission across the three consecutive mating trials within novel partner and familiar partner treatments**. The Box-plot (note logarithmic scale on y-axis) also shows original data points (jittered) and connects the medians per trial with a line to indicate that the analysis was based on repeated measures per individual. For statistics see Table 1.

**Figure 3:**
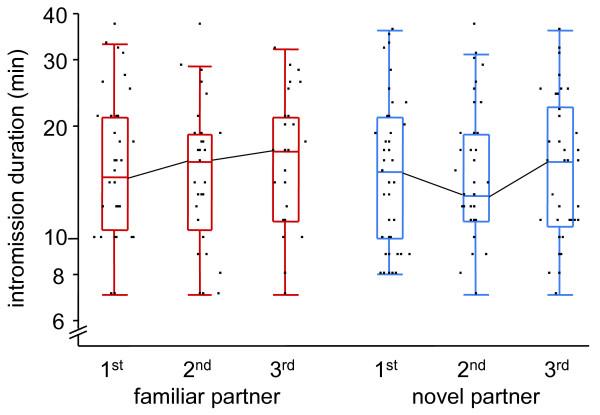
**Penis intromission durations across the three consecutive mating trials within novel and familiar partner treatments**. See Fig. 2 and Table 1 for display and statistics, respectively.

The predicted decrease in sexual motivation in 'familiar partner' focals relative to 'novel partner' focals should manifest in a significant time*treatment interaction in our repeated measures analysis (see Methods for details). Contrary to this expectation, interaction terms were highly insignificant for all our measurements of mating delay and copulation duration (all *P *> 0.29; Table 1; two examples displayed in Figs. 2 and 3). Three measurements (delay until intromission start, delay until intromission end, and duration from first contact to intromission) significantly decreased over time in both treatments, indicating that the overall mating propensity increased over a day.

**Table 1:** Effects of treatment ('novel' vs. 'familiar partner'), time (three successive trials), and their interaction on various measures of mating delay and copulation duration.

**Source**	**df**	**SS**	**F**	***P***	**R^2 ^(adj.)**
Delay until 1st contact					
Model	69	37.743	0.919	0.6459	-0.029
Treatment	1	1.672	3.170	0.0797	
Time	2	1.994	1.676	0.1912	
Time*treatment	2	0.495	0.416	0.6605	
Individual(treatment)	64	33.757	0.887	0.7009	
Error	128	76.160			
Delay until 1st penis eversion					
Model	69	31.732	1.193	0.1946	0.063
Treatment	1	0.340	0.743	0.3919	
Time	2	1.988	2.579	0.0798	
Time*treatment	2	0.358	0.464	0.6299	
Individual(treatment)	64	29.251	1.186	0.2075	
Error	128	49.345			
Delay until intromission start					
Model	69	18.425	1.178	0.2114	0.059
Treatment	1	0.029	0.131	0.7186	
Time	2	3.286	7.250	0.0010	
Time*treatment	2	0.503	1.110	0.3328	
Individual(treatment)	64	14.131	0.974	0.5382	
Error	128	29.009			
Delay until intromission end					
Model	69	12.624	1.292	0.1066	0.093
Treatment	1	0.017	0.107	0.7451	
Time	2	1.940	6.848	0.0015	
Time*treatment	2	0.391	1.382	0.2548	
Individual(treatment)	64	9.956	1.099	0.3231	
Error	130	18.127			
Duration from 1st contact to penis eversion					
Model	69	48.874	0.890	0.6999	-0.040
Treatment	1	0.143	0.192	0.6629	
Time	2	0.838	0.527	0.5918	
Time*treatment	2	0.116	0.073	0.9298	
Individual(treatment)	64	47.790	0.939	0.6053	
Error	127	101.047			
Duration from 1st contact to intromission					
Model	69	28.061	1.368	0.0641	0.114
Treatment	1	0.001	0.002	0.9669	
Time	2	6.946	11.682	<0.0001	
Time*treatment	2	0.621	1.044	0.3549	
Individual(treatment)	64	19.672	1.034	0.4295	
Error	128	38.055			
Intromission duration					
Model	69	13.664	1.187	0.2017	0.061
Treatment	1	0.002	0.008	0.9273	
Time	2	0.104	0.312	0.7326	
Time*treatment	2	0.114	0.340	0.7121	
Individual(treatment)	77	13.473	1.261	0.1342	
Error	128	21.361			

The factor individual (nested within treatment) represents the within subject effect of the repeated measures ANOVA.

### Mate novelty and mating roles

**Figure 4:**
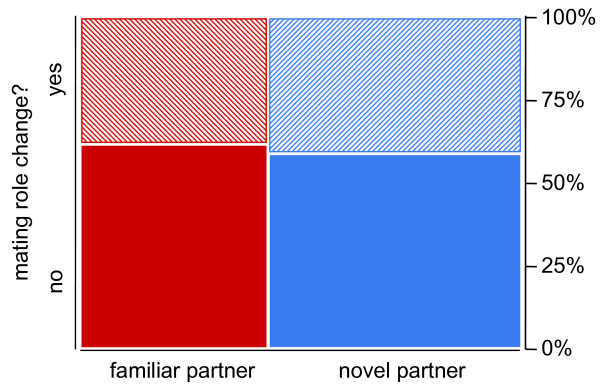
**Mating role changes between a focal snails' first and second mating**. The mosaic plot shows the proportion of focal individuals switching roles split between 'familiar partner' (*n *= 29) and 'novel partner' (*n *= 39) treatment.

We further hypothesised that partner novelty should affect the propensity to change mating roles, such that focals meeting a familiar partner in their second mating should preferentially assume the female mating role, whereas focals meeting a novel partner should assume their mating role at random. In contrast to this prediction, we found random choice of mating roles in both treatment groups ('familiar partner': Likelihood Ratio χ^2 ^= 1.707; df = 1; *P *= 0.19; 'novel partner': Likelihood ratio χ^2 ^= 1.263, df = 1; *P *= 0.26). Consequently, the two treatment groups did not differ in the proportion of focal snails that changed their mating role from first to second mating (Likelihood Ratio χ^2 ^= 0.067; df = 1; *P *= 0.79; Fig. 4) or from second to third mating (Likelihood Ratio χ^2 ^= 0.016; df = 1; *P *= 0.89).

## Discussion

Under our first hypothesis, we assessed the key prediction of the Coolidge effect that focal snails repeatedly exposed to a familiar partner should show a decline in sexual motivation relative to focals exposed to novel partners. Yet, none of our proxies of sexual motivation revealed any divergence in behavioural traits between treatments across the three mating trials. This strongly indicates that, at least within our experimental paradigm, *B. glabrata *does not perform mate discrimination based on partner novelty.

As an ultimate reason, the costs of male matings in *B. glabrata *may not be sufficiently high to select for strategic mate discrimination und prudent mating effort, which may contrast with the pond snail, *Lymnaea stagnalis*, for which an effect of partner familiarity on male mating motivation has recently been demonstrated [25]. Circumstantial support for this hypothesis comes from the observation that mating rates in *B. glabrata *[44] are substantially higher than those found in *L. stagnalis*, where the restoration of seminal fluids may take several days [37]. Hence, male sperm depletion appears to be much less likely in *B. glabrata*, reducing the benefits of prudent mate discrimination.

As a second explanation, the fitness gains from inseminating novel mates may not sufficiently exceed those from repeatedly inseminating the same female. *B. glabrata *snails lay egg masses at a rather high rate (about 1 per day, [50]) such that repeated inseminations of the same female may indeed provide fertilisation chances similar to those of insemination a novel female. On the other hand, however, previous studies show that single ejaculates are sufficient to fertilise consecutive egg masses for several weeks in this species [51,52], rather suggesting low fitness returns for 'topping up' sperm stores in short succession [14]. Measurements revealing the net effect of these opposing effects of multiple mating are necessary to evaluate this hypothesis for an absence of a Coolidge effect in *B. glabrata*.

A third explanation refers to the spatial population composition. A recent study in wild guppies found mate discrimination for partner novelty only in confined populations where individuals could familiarise with each other, whereas discriminatory behaviour did not occur in open river populations [53]. Our study population was long maintained at high population densities, where random mate encounters usually occur with novel partners, perhaps making mate discrimination obsolete. In the natural habitats of *B. glabrata*, founder events and seasonal variation in water flux may occasionally confine small groups to isolated water pools [22]. It is conceivable that such groups may temporarily display discriminatory behaviour conforming to a Coolidge effect, providing a promising avenue for further research.

The absence of a Coolidge effect in *B. glabrata *could also have a proximate basis, where snails may be incapable of using cues that discriminate novel from familiar partners. First, self-referent tags as documented for decorated crickets [29] could be left within the mucus that snails leave on a partner's shell during copulation [25]. *B. glabrata *mucus contains species-specific signals that allow identifying conspecifics [54]. However, even if these cues were informative for individual discrimination, the causative mucus components decay within 10 to 30 *min *[54,55] and would thus only work as a short term self-referent cue. Given a delay between copulations of 1 *h *and more and the long time over which received allosperm can be stored to produce fully fertile egg masses (3 weeks at least, [52,56]), this mechanism appears insufficient to allow partner discrimination in *B. glabrata*. Second, snails may individually recognise previous partners as recently shown for a burying beetle [26]. This would allow discrimination for much longer intervals, but requires snails to process and memorize this more complex information. *B. glabrata *is known to perform sophisticated partner discrimination based on current and potential parasite infections status [57]. Moreover, other pulmonates such as *L. stagnalis *express conditioned behaviour for up to several weeks, indicating long-term memory [58,59]. Yet, a capability of *B. glabrata *to remember individual chemical cues for several hours at least has not been shown to date and may be questionable in the light of our findings.

Part of the discrepancy between our study and the findings in *L. stagnalis *could also be due to differences in the experimental designs. While Koene & Ter Maat [25] initiated their experiment with snails isolated for two weeks, we kept isolation times short to closely mimic the natural conditions under which a biologically relevant Coolidge effect would have to be detectable. Hence, even though small motivational effects may have gone unseen in our study, these are unlikely to affect natural behaviour. Moreover, Koene & Ter Maat [25] found the Coolidge effect most pronounced in snails maintained in their own 'dirty' water. We placed snails into new observation chambers for each mating trial, but allowed them to sequester chemical compounds and mucus for 1 *h *prior to mating trials. This ensured that chemical cues for partner discrimination (e.g. waterborne signals or mucus trails) could still be delivered, but simultaneously avoided that treatments differed in a second factor: if focal snails are kept in their original aquaria, both snails in 'familiar partner' replicates always experience a known environment, whereas the changing partners in 'novel partner' replicates are confronted with a new environment in each mating trial.

Our second hypothesis predicted that focal snails, having all mated in the male role in their first mating, should preferentially assume the female role when encountering the same partner a second time. In contrast, mating roles should be taken at random when the second mate is novel. Contrary to this prediction, the observed changes in mating roles between first and second mating trial did not deviate from the random 1:1 distribution in either group. Moreover, when testing the two experimental groups directly against each other, we found no difference in the frequency of mating role change to the second or to the third mating. These findings indicate that, independent of mate novelty, previous mating history does not affect the subsequent choice of mating roles in *B. glabrata*. These conclusions complement an earlier observational study that documented random alternation of mating roles and no preference for one particular mating role in snails that were kept in larger groups [44]. It is known that mating roles in *B. glabrata *copulations are determined very early during courtship where the active snail will later take the male role [42]. Yet, to date, the ultimate and proximate factors that determine these activity levels remain obscure.

## Conclusion

Our study shows that effects of mate novelty on sexual motivation may be less prevalent than previously thought. Candidate reasons for the absence of a Coolidge effect in *B. glabrata *include a low benefit to cost ratio of discriminatory behaviour as well as the absence of the required proximate mechanisms to distinguish novel from familiar partners. Disentangling these alternatives will be an important goal for further research. We further find no evidence for the novel hypothesis that sex-specific familiarity affects the choice of mating roles in this simultaneous hermaphrodite. Future work should address this idea in further systems that represent a broader range of ecological and social contexts to elucidate the generality of our findings.

## Methods

### Experimental groups and sample sizes

Our experiment contained two treatments. Treatment 1 ('*familiar partner*') served as our baseline control. Here, focal individuals were repeatedly exposed to a familiar partner during three successive mating trials. In treatment 2 ('*novel partner*') snails were confronted with a novel partner in each of the three trials (Fig. 1). With a Coolidge effect present, we expected 'novel partner' focals to show lower rejection rates, shorter mating delays and/or longer copulations than 'familiar partner' focals.

In total we initiated 40 replicates (80 snails) in the 'familiar partner' treatment and 56 replicates (112 snails) in the 'novel partner' treatment. This difference in initial sample size was necessary because, in order to exclusively test for the effect of mate novelty, we had to standardise the history of previous mating roles in the 'novel partner' treatment. In the 'familiar partner' treatment, previous mating roles of both snails per replicate are automatically complementary across all three mating trials. In the 'novel partner' treatment, we therefore had to similarly assure that focal snails obtained a partner with a complementary mating history. For example, a focal snail that had mated in the male role in both its first and second mating must - for its third mating - be exposed to a 'novel partner' that had previously mated twice in the female role. The initially larger number of replicates in this treatment group buffered the expected loss of focal snails for which we could not provide complementary partners in all three mating trials.

### Experimental procedure

Our experimental snails originated from a large laboratory stock population (N ~ 500) that has been kept at constant water temperature (26°C) and a 12 *h*:12 *h *light:dark cycle for several dozen generations. Prior to the experiment, 150 snails each were placed in two 60 l freshwater tanks. This assured that we were measuring the relevance of the Coolidge effect under high density conditions as often encountered in the field, and excluded the possibly confounding effects of an elevated mating propensity when encountering virgin partners [60].

As it was impossible to simultaneously observe all 96 replicate pairs, we spread our experiment across four consecutive observation days (experimental runs). During each run we observed 10 'familiar partner' and 14 'novel partner' replicates in parallel. In order to simulate the high rate at which *B. glabrata *typically meet (at least 4 matings in 12 *h*, [44]), all three mating trials per replicate were performed within a 12 *h *period. The following describes the experimental procedure for a single experimental run (Fig. 1), which was identically repeated for the remaining three runs.

60 *h *prior to observation, we randomly selected 48 mature snails and placed them in groups of six in 1.5 l PE-boxes. The six snails per box were isolated by perforated plastic walls but shared the same water to perceive the presence of other snails [61]. 42 *h *prior to observation, snails were fed with organic lettuce *ad libitum*. Since body size may affect mating behaviour, snails were allocated to treatment groups randomly with respect to body size, but we size-matched partners within allocated pairs as closely as possible. Mean shell diameter of all experimental snails was 1.63 cm ± 0.26 cm SD (range 1.0 cm to 2.1 cm). Diameters did not vary between our experimental groups both overall and within each observation day (all ANOVA *P *> 0.25). The two snails per pair were individually marked with orange and green paint markers to enable individual recognition. In a related freshwater snail, *Physa acuta*, these paints do not affect individual condition [62] and accordingly showed no effect on mating role choice in our first mating trial (Likelihood Ratio χ^2 ^= 0.114; df = 1; *P *= 0.74).

The observation day (consisting of the three consecutive mating trials) started with the light phase. The two snails per allocated pair were placed in a transparent, rectangular plastic box with 190 ml fresh water at 26°C. Individuals remained separated for 1 *h *by a perforated transparent partition to allow mucus secretion and visual as well as chemical communication but prevent direct contact. Thereafter, both individuals were briefly lifted out of the container and placed randomly together in one of the two compartments. We now recorded the behaviour of all snails for 3 *h *(N = 24 pairs). Following a successful mating, pairs were immediately separated and returned to their former compartment for the remaining observation period. Pairs were not allowed to mate reciprocally and were separated if necessary. We only counted penis intromissions exceeding 5 *min *as successful copulations [42]. Snails that were not mating or probing were separated after 2 *h *and 50 *min*. Pairs that did not copulate during the first mating trial were excluded from the experiment.

After this first mating, the individual performing the male role was determined 'focal' for all consecutive trials and subsequent analyses. Focals were now additionally marked with a white dot (Fig. 1).

Handling procedures for the second and third mating trials per run were almost identical (Fig. 1). Snails were first transferred into new compartmented boxes with fresh water. 'Familiar partner' focals were paired to their previous mating partner. 'Novel partner' focals obtained an unfamiliar partner with a complementary mating history (as outlined above). These changing partners randomly rotated between pairs of the second treatment. Following the initial 1 *h *of separation (again allowing mucus secretion and chemical communication), both snails were lifted again, and this time always placed in the compartment of the novel or familiar partner.

### Measurements

As proxies of sexual motivation, we recorded in each trial (i) whether mating took place or not, (ii) the mating roles taken, (iii) copulation latency, and (iv) copulation duration. Copulation latency was approximated by multiple alternative measures: delay until first contact; delay until first penis eversion; delay until intromission start and end; time lag between first body contact and penis eversion; time lag between first body contact and start and end of intromission. Copulation duration may not necessarily reflect the amount of semen transferred, but for the purpose of our study we consider shorter copulations a useful proxy of declining sexual motivation. Copulation duration was approximated by the time interval between penis intromission and retraction. Successful penis intromission into the partner's gonopore was reliably indicated with the penis reaching under the partner's frontal left shell opening without further movements (unpubl. data).

### Analyses

We used repeated measures ANOVAs to assess the differential effects of treatment on all continuous (time) measurements across the three consecutive mating trials (i.e., the three repeated measures per focal). In these analyses, a Coolidge effect would become visible in a significant interaction between the fixed factors *treatment *and *mating trial*. For example, 'familiar partner' focal snails should show a steeper increase in mating delay than 'novel partner' focals that maintained sexual motivation. Model parameters were derived from a two-way nested ANOVA, with treatment and mating trial as fixed factors and individual ID as a factor nested within treatment [63]. Original data varied neither between observers nor between experimental runs, such that both these factors where omitted from the final reported analyses. After log-transforming all continuous variables, none of these violated the RM-ANOVA assumption of sphericity [63]. We therefore only report the uncorrected results. After excluding dead snails and their partners, non-mating pairs in the first mating trial, or 'novel partner' focals for which we could not find a mate with complementary history, all RM ANOVAs are based on *n *= 28 focal snails in the 'familiar partner' treatment and *n *= 38 focal snails in the 'novel partner' treatment.

Changes in the mating roles of focal individuals were analysed using a two dimensional frequency test. We first compared the two treatments between first and second mating trial ('familiar partner' treatment *n *= 29; 'novel partner' treatment *n *= 39) and between second and third mating trial ('familiar partner' treatment *n *= 28; 'novel partner' treatment *n *= 38). Second, we tested separately for each treatment whether the proportion of focals mating in the male or female role deviated from the random 1:1 expectation.

All data analyses were performed using JMP 7.0.2 statistical package (SAS Inc.).

## Competing interests

The authors declare that they have no competing interests.

## Authors' contributions

IH and JW carried out the study, participated in its design and analysis, and drafted the manuscript. NM and NT contributed to the study design and manuscript preparation. NA coordinated study design, statistical analysis, and manuscript preparation. All authors read and approved the final manuscript.
